# Progress in Neurology

**Published:** 1898-07-16

**Authors:** 


### Progress in Neurology.

(*Continued from page* 256.)

**Acute Ataxy of One Limb.**—Thomson[14] has recorded two cases of ataxy in one limb only, of acute onset. The condition is a rare one, and when resulting from organic disease of the cord is probably in most cases due to a focal lesion situated somewhere in the posterior columns, so that the muscular sense impressions are interrupted. The ataxy is usually accompanied by complete muscular anæsthesia, so that all power of estimating weights is lost, though the cutaneous sensibility may be perfect.

**Hereditary Spastic Paraplegia** has been known for some time, but recently Bayley[15] has brought forward conclusive proof of its existence in his record of a family in which five successive generations have been affected by the disease, which is characterised by a spastic condition, coming on as a rule about the fifth or sixth year, associated with exaggeration of knee-jerks, and usually also ankle-clonus, and without any sensory impairment or pupil affection.

Starr[16] contributes an interesting paper on some unusual forms of apoplectic attack. He remarks that a hemiplegia is so commonly a sequel of an apoplexy that one is inclined to think the association is inevitable. It is frequent, no doubt, by reason of the fact that hæmorrhage and embolism are far more common in the domain of the middle cerebral than in that of the other blood vessels. If, however, some other vessel, *e.g.*, the anterior or posterior cerebral, is obstructed or ruptured, there is not necessarily any subsequent paralysis. But the local symptoms will vary according to the region of brain affected, and careful attention to the symptoms will enable a diagnosis to be made in many cases. In four cases in which the lesion was probably in the region of the anterior cerebral, there was sudden severe pain in the frontal region on the side of the lesion, slight confusion of speech and slight temporary facial paresis, and distinct mental symptoms. These mental symptoms, due to the damage to the frontal lobes, were mental confusion and dulness, with inability to concentrate the attention and to exercise self-control, particularly over the expression of the emotions. In two cases where the posterior cerebral artery was affected the most prominent after-feature was a hemianopsia towards the side opposite the lesion.

In two cases where the lesion was in all probability cerebellar the initial symptoms were the sudden onset of vertigo, headache, vomiting, and prostration, from the latter of which the patients slowly recovered in some weeks, but the vertigo persisted. It was absent whilst the patients were sitting or lying down, but any attempt at standing or walking produced it, and an ataxy of cerebellar type accompanied it. There were no aural symptoms.

In the treatment of such cases it is important to take all precautions against effort of mind or body, exactly as in the case of hemiplegia, so as to allow of repair of the vascular lesion as far as that is practicable.

**Epidemic Cerebro-Spinal Meningitis.** — Councilmen Mallory and Wright[17] contribute an elaborate paper on the pathological anatomy of over a hundred cases of epidemic cerebro-spinal meningitis which occurred in Boston during last winter and spring, and in which the mortality was 68 per cent. In the majority of cases the diplococcus of Weichselbaum was the organism found; this was almost strictly confined to the interior of the polynuclear leucocytes and never found in the bodies of other cells. In no case were the diplococci found except in connection with the lesions of the disease; it was absent from cultures of the blood, liver spleen, and kidneys. Mixed infection with other organisms was not uncommon, *e.g.*, with the pneumococcus the diplococci were found by lumbar puncture in 70 per cent. of the cases examined by this method. Meningitis apart from the epidemic form was found to be most commonly caused by the tubercle bacillus, the pneumococcus and the streptococcus. In most of these cases the meningitis was secondary to infection elsewhere. The difference between the clinical history of pneumococcus meningitis as compared with the epidemic form is in the absence or slight development of symptoms in the former condition, and in the extension of infection along the cranial nerves.

Acute meningitis may be produced by the entrance into the meninges of a number of infectious organisms. These forms are rarely primary. The organisms enter the meninges either by the formation of a communication between the meninges and some cavity where they may be accidently present (as in the middle ear or nose), or by the extension to the meninges of an infectious process in the vicinity (mastoiditis, erysipelas), or they are brought to the meninges by the blood from some other focus in the body (pneumonia, endocarditis). In tuberculous meningitis no case was found in which the lesion in the meninges could be regarded as primary. The only two cases of apparently primary infection were due to the pneumococcus, and in one of these the infection may have come from the intestinal canal. Probably all infections of the meninges, other than the diplococcus of Weichelselbaum, are fatal, but this can only be determined by examination of exudatum obtained during life by spinal puncture. If tubercle bacilli, pneumococci, or streptococci are found with evidences of meningitis in a case which recovers, it would settle the point; clinical evidence without lumbar puncture will not.

**Meningitis.**—Wolf[18] has investigated the etiology of meningitis arising from old middle-ear disease from a study of the literature of the subject. The results showed that in 86 per cent. of the cases the pneumococcus, or the meningococcus intracellularis, or the streptococcus was the exciting agent. It is thus clear that purulent meningitis is not necessarily the result of infection by any one species of bacteria; but that a considerable number are capable of acting as its cause.

Von Ranke[19] has employed lumbar puncture in 25 cases of meningitis, 19 of which were of tuberculous nature. In no case as yet has it produced a cure. All the tuberculous cases died. Instances occurred in which a temporary improvement followed puncture, but in most cases there was no change in the condition of the patient. The cases in which some temporary improvement followed were in an early stage, when the pressure had not lasted long. It is in the cases of tuberculous meningitis, in which the diagnosis from the clinical signs is doubtful, that lumbar puncture was useful. The number of tubercle bacilli in the fluid was small; the fluid was clear and usually colourless or slightly green or yellow. The amount of fluid drawn off was usually from 20 to 30 c.cm., and the pressure high from 160 to 300 mm. of water. No harm of any kind was produced by the puncture.

[14] Thomson, Lancet, Dec. 18, 1897. [15] Bayley, Amer. Jour. Nerv. Dis., Nov., 1897. [16] Starr. [17] Councilman, Mallory, and Wright, Amer. Jour. Med. Sci., Feb., 1898. [18] Wolf, Berl. Klin. Wochen, No. 10, 1897. [19] Von Ranke, Münch Med. Woch., Sept. 21, 1897.

